# Mental Contrasting of a Negative Future with a Positive Reality Regulates State Anxiety

**DOI:** 10.3389/fpsyg.2017.01596

**Published:** 2017-09-20

**Authors:** Gunnar Brodersen, Gabriele Oettingen

**Affiliations:** ^1^Department of Psychology, University of Hamburg Hamburg, Germany; ^2^Department of Psychology, New York University, New York NY, United States

**Keywords:** self-regulation, thinking about the future, anxiety, mental contrasting, fantasies

## Abstract

Mental contrasting of a desired future with impeding reality is a self-regulatory strategy fostering goal pursuit. However, there is little research on mental contrasting of a negative future with a positive reality. We conducted two experiments, each with four experimental conditions, investigating the effects of mental contrasting a negative future with a positive reality on state anxiety: participants who mentally contrasted a negative future regarding a bacterial epidemic (Study 1, *N* = 199) or an idiosyncratic negative event (Study 2, *N* = 206) showed less state anxiety than participants who imagined the negative future only or who reverse contrasted; participants who mentally elaborated on the positive reality also showed less state anxiety. Our findings suggest that mental contrasting of a negative future helps people reduce disproportional anxiety regarding a negative future.

## Introduction

Whether it be an important job interview, an exam, or a flight over the Atlantic: From time to time, we all find ourselves facing a future event in our everyday lives that may evoke fear or anxiety. Even if the likelihood of potential harm is very low, our thoughts revolve around this event, getting us worried and tense. Very rarely, it is reasonable to give in to our anxieties. Rather, in most cases overcoming our fears and approaching the future will be more effective for our well-being. In other words, we need to cope with our fears about the future. However, people often draw on passive coping strategies such as avoidance or waiting instead of actively regulating their fears (Thayer et al., 1994). We investigated to what extent the self-regulatory strategy of mental contrasting (Oettingen, 2000, 2012) can help people to regulate their fears. Whereas most research on mental contrasting refers to mentally contrasting a positive future with the negative reality standing in the way of reaching the future, we had people mentally contrast a negative future with the positive reality. Specifically, we hypothesized that mental contrasting of a negative feared future enables people to reduce their state of anxiety regarding that negative future.

## Fears and Anxiety

Fears in everyday life are characterized by an anticipation of negative events in the near or far future (Hoerger et al., 2012). Thereby, fear and anxiety are often used interchangeably to describe the response to the same anticipated negative future stimulus or event. Indeed, fear and anxiety are closely related and overlapping concepts. Both fear and anxiety include an aversive feeling and bodily tension, related to threat (Öhman, 2008). Epstein (1972) argued that fear refers to the organism’s attempt to cope with perceived danger, especially by avoidant behavior or flight. If a fear remains unresolved (i.e., coping attempts fail), it turns into anxiety. Anxiety in turn is a specific emotional state of perceived threat and leads to a shift in cognitive and physiological processes (Bijsterbosch et al., 2014). More specifically, anxiety is characterized by multidimensional symptoms that can be assigned to a cognitive anxiety component (including worry, a lack of concentration, and other cognitive impairments) and a somatic anxiety component (including bodily tension, sweat, and other physiological shifts; Liebert and Morris, 1967; Davidson and Schwartz, 1976). Anxiety can be measured as (1) a present feeling in a given situation (i.e., state anxiety) or (2) as an individual’s stable tendency to react across a variety of situations (i.e., trait anxiety; Spielberger, 1966). In our research, we sought to investigate the effects of mental contrasting of a specific negative future on the subsequently arising emotion of anxiety regarding that negative future. Consequently, we focused on state anxiety as our dependent variable. However, we assessed participants’ baseline trait anxiety as a control variable.

Scientists agree that anxiety plays an important role in evolution: it activates the autonomic nervous system and thus prepares the organism to react appropriately to threat. In other words, it is an essential part of the organism’s defense system (Öhman, 2008). However, in many scenarios responses of anxiety are not proportional to the actual probability of the feared future event occurring. Then they are considered problematic in that they prevent people from dealing constructively with the situation and also with related future events (Rapee, 1991). Thus, their behavior may be determined by emotion-based thoughts and feelings rather than by problem-focused activities (Welch et al., 2009).

Based on these considerations, we talk about fear of future events or scenarios (e.g., fear of *Escherichia coli*). We refer to fear as a fear that is considered as not proportional to the actual probability of being harmed by the respective future event. Regarding the assessment of people’s emotional state in response to fear about future events we refer to anxiety and therefore to anxiety regulation. An emotion like fear or anxiety can be characterized by its motivational function. Brehm (1999) even postulates that emotions are motivational states. As a consequence, Brehm argued that the intensity of an emotion can be seen as a result of “the difficulty of attaining the goal of the emotion.” (Brehm, 1999, p. 4). If the goal is easily attainable or completely out of reach, the intensity of emotion is said to be low; on the contrary, if the goal is difficult to attain, the intensity of emotion is said to be high. A central aspect of these considerations is that the “function of emotions is to urge behavior designed to promote or prevent important potential outcomes” (Brehm, 1999, p. 4). Accordingly, we suggest that the reduction of state anxiety would influence people’s motivation and behavior as well. When people’s fear of a negative future decreases, they will be less anxious and less likely to avoid that negative future. For example, people who reduce their fear of *E. coli* would be willing to invest less into campaigns of *E. coli* prevention, but more into alternative campaigns.

### The Regulation of Anxiety

We wanted to investigate if mental contrasting of a negative future can be used to regulate state anxiety regarding that negative future. Emotion regulation in general refers to “the processes by which we influence which emotions we have, when we have them, and how we experience and express these emotions […]” (Gross, 2008, p. 500). With regard to anxiety, people use different strategies to reduce its extent. Gross and Thompson (2007) postulated a process model of emotion regulation and highlighted that one may address one of five stages in the emotion regulation process. To summarize, one may (1) choose or (2) modify the particular situation; people may also (3) redirect their attention (e.g., by distraction) or (4) change the subjective meaning of the situation (e.g., by reappraisal); and finally, one may (5) directly modulate the emotional response (e.g., by relaxation).

However, in everyday life it is often not feasible to use one of these approaches, or it is too costly. For example, people may be unable to freely choose or modify a situation. Further, changing the meaning of a situation requires that a person is able to think about alternative meanings, which is especially difficult if the anxiety-evoking event is in the future. Distraction might be too costly because it has problematic long-term effects regarding physiological arousal and stress (John and Gross, 2004; Gross and Thompson, 2007). Finally, the modulation of the emotional response is costly as it is very late in the regulation process; people might benefit from starting with emotion regulation before facing the anticipated negative event. To sum up, people’s habitual emotion regulation strategies may not be suited to a fear evoking situation, or they may have maladaptive consequences such as distraction, avoidance, or suppression (Hayes et al., 1999; Wenzlaff and Wegner, 2000; Hayes et al., 2004). Thus, people should benefit from a strategy that allows active coping and directly addresses the individual’s feared future so that the fear is unveiled as unreasonable or not proportional to the actual probability of being harmed. Mental contrasting is such a strategy. Therefore, we assume that people may benefit from mental contrasting of a negative future to attenuate anxiety elicited by a feared future.

## Mental Contrasting

Mental contrasting (Oettingen, 2000, 2012) is a self-regulatory strategy that fosters selective goal pursuit. In mental contrasting, people start by mentally elaborating on a desired future (e.g., pass my exam successfully) and subsequently reflect on the present reality standing in the way of that desired future (e.g., partying). As a result, expectations of success (i.e., how likely it is that the person can attain the desired future) are activated which in turn leads to expectancy-dependent goal pursuit: thus, when expectations of successfully realizing the wish are high, people will strongly commit and invest into attaining the desired future. Research on mental contrasting provides evidence for its beneficial effects on goal pursuit and behavior change across life domains, age, and culture [see Oettingen (2012, 2014) for overviews].

### Mental Contrasting and Anxiety

Most research on mental contrasting refers to a desired (i.e., positive) future. However, fear refers to an undesired (i.e., negative) future. Specifically, feelings of anxiety result from anticipating a feared future. We therefore investigated the effects of negative thoughts and images about a feared future contrasted with the present reality standing against the negative future coming true. Indeed, there is empirical evidence on mental contrasting of a negative future. Oettingen et al. (2005) conducted a study with high school students from former East Berlin. Participants first elaborated on the feared future of foreign youth moving into the neighborhood. They were instructed to think about repercussions they might suffer from the foreign youth moving in and how this change in context might interfere with their everyday lives. Subsequently, participants were randomly assigned to one of three experimental conditions. Participants in the mental contrasting condition, after they had elaborated on the negative future of having to live with the foreign youth, either freely elaborated on statements which the experimenters provided about a positive reality standing in the way of the feared future (e.g., statements about great soccer matches with foreign youth; mental contrasting condition), on the negative future only (negative future condition), or they were guided away from the negative future and reflected on the positive reality only (positive reality condition). The students’ expectations and incentive value to be able to integrate foreign youth were assessed prior to the manipulation. Oettingen et al. (2005) found that even 2 weeks after the experiment, participants who had high expectations showed a greater tolerance toward foreign youth and were more willing to invest time and effort in integrating them. To summarize, mental contrasting of a negative future helped participants approach that future.

In the present paper, we sought to extend this finding by investigating the effects of mental contrasting of a negative future on the regulation of the emotional state of anxiety regarding that negative future. More specifically, based on the finding that mental contrasting enabled youth to approach their feared future, we hypothesized that it might attenuate the anxiety that arises from a present fear that is perceived as out of proportion: when chances of a negative future coming true are low, mental contrasting makes these low chances salient and thereby leads to a reduction of anxiety. In other words, by mentally contrasting a negative future with a positive present reality standing in the way of that future, people should realize that their fear is not in proportion with the actual threat. As a consequence, state anxiety should decrease.

A further question was whether exposing people to a specific fear, without explicit mental elaboration of the feared future, would produce enough salience of the feared future to elicit mental contrasting effects. We ask this question, because as outlined above, a feared future is automatically associated with the organism’s defense system, a negative future might be more salient than a positive future. Further, as Taylor (1991) pointed out, negative events elicit “more physiological, affective, cognitive, and behavioral activity […] than neutral or positive events” (p. 67). As a consequence, putting the positive reality against the negative future in the positive reality condition may be comparable to the procedure in the mental contrasting condition. If so, it would be enough to induce the positive reality to achieve mental contrasting effects on anxiety reduction. To explore this idea, we specifically included a control condition in which participants had to only elaborate the positive reality.

## The Present Research

In the present research, we investigated the extent to which mental contrasting of a negative future attenuates state anxiety vis-a-vis the specific fear-eliciting future event. Specifically, we focused on state anxiety as a result of fears that prevented people from constructively dealing with their feared future. To address this research question, we conducted two studies in which participants should apply one of four strategies: (1) participants in the mental contrasting condition were instructed to mentally elaborate on both their feared future and the positive reality standing in the way of their feared future coming true; (2) participants in a negative future condition should only mentally elaborate on their feared future; (3) participants in a positive reality condition should only mentally elaborate on the positive reality standing in the way of their feared future; and (4) participants in a reverse contrasting condition should mentally elaborate on both their feared future and the positive reality; however, unlike in the mental contrasting condition, participants in the reverse contrasting condition started with the positive reality.

We included a reverse contrasting condition because it is the most critical control condition. In fact, it resembles the mental contrasting condition in all aspects other than the order of elaboration of future and reality: In the reverse contrasting condition, the positive reality is elaborated before the feared future. With regard to mental contrasting of a positive future, it has been extensively shown that the critical mechanisms leading to changed behavior occur only after mental contrasting, but not after reverse contrasting. Specifically, a strong mental association between future and reality, between reality and the means to overcome the reality as well as the interpretation of the reality as an obstacle only occur after mental contrasting (Kappes et al., 2012, 2013; Kappes and Oettingen, 2014). Therefore, we hypothesized that in the reverse contrasting condition participants should not be able to effectively regulate their anxiety in response to a feared future.

In Study 1, we investigated the effects of mental contrasting on anxiety arising from the fear of a bacterial epidemic of *E. coli*. In Study 2, we sought to extend the findings of Study 1 and investigated the effects of mental contrasting on state anxiety arising from an idiosyncratic fear.

## Study 1: Fear of *E. coli*

We sought to investigate the effects of mental contrasting of a negative future on a specific fear, namely the fear of a bacterial epidemic caused by *E. coli*. *E. coli* (i.e., Enterohemorrhagic *Escherichia coli*) is a bacterium that has some pathogenic serotypes which may cause the so-called hemolytic–uremic syndrome with severe symptoms such as bloody diarrhea and renal failure. The ethical review committee of the University of Hamburg and New York University approved the procedure and materials. In the beginning of the study we had participants read an informational article about *E. coli* including both, facts about the disease and statements which indicate that in the United States it was very unlikely that an epidemic would occur. Then participants were randomly assigned to the mental contrasting condition, the negative future condition, the positive reality condition, or the reverse contrasting condition. As a dependent variable, we assessed self-reported state anxiety. Participants in the mental contrasting condition should realize that their fear is not proportional to the likelihood of the negative event occurring and thus show less state anxiety than in the three control conditions. The study was designed as an online study, using a one-factorial (self-regulatory strategy: mental contrasting vs. negative future vs. positive reality vs. reverse contrasting) design.

### Method

#### Participants

Power analysis for a one-way ANOVA with four groups was conducted to determine a sufficient sample size using an alpha of 0.05, a power of 0.80, and a medium effect size ($\eta_{p}^{2}$ = 0.06; Faul et al., 2013). Based on the aforementioned assumptions, the desired sample size was identified as 180. To account for potential study dropouts, we recruited 214 participants via Amazon Mechanical Turk (MTurk). To ensure data quality, we only recruited participants who had above 95% approval ratings on MTurk. This procedure is in line with Peer et al. (2014). Fifteen participants were excluded from data analyses because they did not mentally elaborate and write down their free thoughts and images during the induction of the self-regulatory strategies. Drop-outs were independent from conditions. The remaining participants (*N* = 199) were included in the data analyses. All participants were living in the United States of America. Forty-nine percent (*n* = 97) were female, age ranged from 18 to 73 years (*M* = 36.21, *SD* = 12.61).

#### Procedure and Materials

Participants were informed about the procedure of the study and signed the consent form. After participation which took about 20 min participants were fully debriefed and received credit for their participation.

#### Information about *E. coli*

To inform participants about *E. coli*, we asked them to read a bogus newspaper article. Importantly, this article included both facts about the *E. coli* bacterial disease (e.g., that humans might get infected even via vegetable food like gherkins or tomatoes) and reasons why an *E. coli* epidemic in the United States should be unlikely to occur (e.g., the hygiene provisions in the United States meet the highest standards which reduces the risks of an infection dramatically). Thus, we made sure that participants understood that the likelihood of getting infected would be very low.

#### Manipulation of the Self-Regulatory Strategy

Participants were randomly assigned to one of four experimental conditions: in (1) the mental contrasting condition, participants were instructed to vividly imagine both the negative future of an *E. coli* epidemic in the United States and the positive reality that was standing against the feared future coming true. Participants should begin with naming the most important aspect of the negative future (“What is the worst thing that you associate with *E. coli* coming to the United States? Identify the worst thing and write it down.”) Then they were instructed to mentally elaborate on this aspect thoroughly. Participants read:

Now really think about this worst thing. Imagine the relevant events and scenarios as vividly as possible. Let your mind go! Do not hesitate to give your thoughts and images free rein. Take as much time and space as you need to write down your thoughts.

After elaborating on the aspect of the negative future, participants should name the most important aspect of the present reality that was standing against the spreading of *E. coli* to the United States. The instructions read:

Sometimes things do not happen although we are afraid they could. What is the most important aspect of the present reality that is standing against the spreading of *E. coli* to the United States? What should hinder the spreading of *E. coli*? Identify the most important thing that should hinder the spreading of *E. coli* to the United States and write it down.

Again, they were asked to elaborate on this aspect. In (2) the negative future condition, participants were instructed to think about the negative future of a spreading of *E. coli*. They were asked to name and mentally elaborate on the worst and the second worst thing they associated with a spreading of *E. coli*. In (3) the positive reality condition, we instructed participants to only think about the positive reality. They were asked to name and mentally elaborate on the most important and the second most important aspects that were standing against a spreading of *E. coli* to the United States. In (4) the reverse contrasting condition, participants were asked to think about both the negative future and the positive reality; however, unlike in the mental contrasting condition, participants in the reverse contrasting condition should begin with the positive reality aspect and only thereafter they are instructed to think about the negative future.

#### State Anxiety

After the manipulation, we assessed the dependent variable, state anxiety using the state anxiety subtest of the *State–Trait-Anxiety Inventory* (*STAI*; Spielberger et al., 1970), adjusting its instructions to fit the topic of *E. coli*. Specifically, we asked participants: “When you think about *E. coli*: how do you feel now?” We then listed the 20 items of the state anxiety subtest of the STAI (STAI-S; e.g., “I am worried”) using a 4-point response scale from 1 (*not at all*) to 4 (*very much so*). Internal consistency was high, Cronbach’s α = 0.95.

#### Control Variables

To be able to statistically control for participants’ general tendency to react with fearful thoughts and feelings, we assessed trait anxiety in the very beginning of the study, using the trait anxiety subtest of the STAI (STAI-T; Spielberger et al., 1970). It also consists of 20 items (e.g., “I feel nervous and restless”) using a 4-point Likert response scale from 1 (*not at all*) to 4 (*very much so*). Internal consistency was high, Cronbach’s α = 0.94. In addition, right after the newspaper article, we assessed a short baseline measure of participants’ state anxiety. Specifically, we used three single items of the STAI-S (e.g., “I feel nervous”) providing high internal consistency, Cronbach’s α = 0.83. Then we assessed participants’ expectations and their incentive value to overcome their fear; specifically, we adapted the instructions of prior research on mental contrasting (e.g., Oettingen et al., 2001) and asked participants: “How likely do you think it is that you will be able to overcome your fear?” and “How important is it to you to overcome your fear?”, both items using a 7-point scale from 1 (*not at all*) to 7 (*very much so*). Finally, we assessed demographic data from our participants like gender, age, profession, and place of residence.

### Results

#### Descriptive Data

We calculated sum scores for baseline trait anxiety (with a maximum possible score of 80) and baseline state anxiety (with a maximum possible score of 12). Participants had a moderate extent of trait anxiety (*M* = 37.92, *SD* = 11.43) and a moderate extent of baseline state anxiety (*M* = 5.40, *SD* = 2.22). When taking the means instead of the sum scores, the scores of trait anxiety (37.92/20 = 1.9), baseline state anxiety (5.4/3 = 1.8), and state anxiety as the dependent measure (39.59/20 = 1.98) were all at comparable levels. Expectations and incentive value to overcome the fear were above the midpoint of the 7-point scale (expectations: *M* = 5.86, *SD* = 1.48; incentive value: *M* = 4.98, *SD* = 1.75).

#### State Anxiety

We computed a general linear model (GLM) with state anxiety as the dependent variable and condition as the categorical independent variable; we included trait anxiety, baseline state anxiety, expectations, and incentive as continuous covariates. We found significant effects for the covariates trait anxiety, *F*(1,191) = 23.37, *p* < 0.001, $\eta_{p}^{2}$ = 0.109 and baseline state anxiety, *F*(1,191) = 63.91, *p* < 0.001, $\eta_{p}^{2}$ = 0.251. More importantly, we also found the expected main effect of condition, *F*(3,191) = 6.97, *p* < 0.001, $\eta_{p}^{2}$ = 0.10.

**FIGURE 1:**
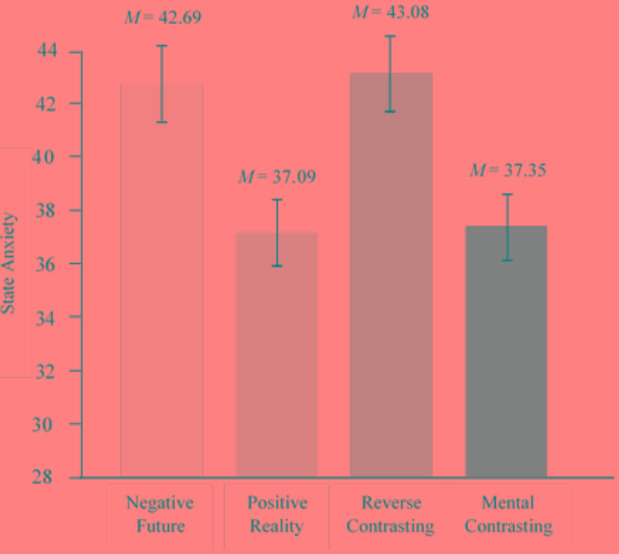
Effects of self-regulatory strategies on state anxiety vis-à-vis the fear of a future *E. coli* epidemic. Error bars represent standard errors. Covariates appearing in the model are evaluated at the following values: Trait anxiety, *M* = 37.92; baseline state anxiety, *M* = 5.40; expectations, *M* = 5.86; and incentive value, *M* = 4.98.

Thus, depending on condition, participants experienced a different extent of state anxiety. Participants who mentally contrasted the negative future with the present reality (*M* = 37.35, *SE* = 1.22, 95% CI [34.95, 39.75]) or only reflected on the positive reality (*M* = 37.09, *SE* = 1.24, 95% CI [34.65, 39.53]) had the lowest state anxiety scores, whereas participants who only reflected on the negative future (*M* = 42.69, *SE* = 1.43, 95% CI [39.87, 45.51]) or reverse contrasted (*M* = 43.80, *SE* = 1.22, 95% CI [34,95, 39.75]) had the highest state anxiety scores, see **Figure 1**.

Pairwise comparisons revealed (1) significantly less state anxiety in the mental contrasting condition, than in the negative future condition, *t*(191) = 2.84, *p* = 0.005, $\eta_{p}^{2}$ = 0.041, 95% CI [-9.05, -1.64], and (2) significantly less state anxiety in the mental contrasting condition than in the reverse contrasting condition, *t*(191) = 3.45, *p* = 0.001, $\eta_{p}^{2}$ = 0.059, 95% CI [-10.14, -2.76]. Remarkably, we found no differences between the mental contrasting condition and the positive reality condition, *t*(191) = 0.15, *p* = 0.881, $\eta_{p}^{2}$ = 0.00, 95% CI [-3.16, 3.68]. In fact, just like the mental contrasting condition, the positive reality condition showed significantly lower anxiety scores than the negative future condition, *t*(191) = 2.96, *p* = 0.003, $\eta_{p}^{2}$ = 0.044, 95% CI [-9.33, -1.87] and the reverse contrasting condition, *t*(191) = 3.57, *p* < 0.001, $\eta_{p}^{2}$ = 0.062, 95% CI [-10.42, -3.00].

An additional GLM without including covariates showed the same results, *F*(3,195) = 5.76, *p* = 0.001, $\eta_{p}^{2}$ = 0.081. Pairwise comparisons revealed (1) significantly less state anxiety in the mental contrasting condition, than in the negative future condition, *t*(195) = 2.85, *p* = 0.005, $\eta_{p}^{2}$ = 0.04, 95% CI [-11.57, -2.12], and (2) significantly less state anxiety in the mental contrasting condition than in the reverse contrasting condition, *t*(195) = 2.86, *p* = 0.005, $\eta_{p}^{2}$ = 0.04, 95% CI [-11.49, -2.11]. There were no differences between the mental contrasting condition and the positive reality condition, *t*(195) = 0.20, *p* = 0.841, $\eta_{p}^{2}$ = 0.00, 95% CI [-3.93, 4.82].

To test whether there were any interaction effects between condition and one of the covariates, we ran four additional GLMs and included the interaction terms between condition and one of the covariates in each of them. There were no significant interaction effects between condition and any of the covariates, all *p*s > 0.098.^1^

### Discussion

Participants who mentally contrasted a negative future of a bacterial epidemic with a positive reality exhibited less state anxiety regarding that negative future than participants who either reverse contrasted, or imagined the negative future only. This effect of condition was of a medium effect size. Thus, our findings suggest that the self-regulatory strategy of mental contrasting of a negative future with a positive reality can be used to regulate present negative emotions in response to a feared future scenario. More specifically, when people mentally contrasted their negative future with positive aspects of the present reality, they down-regulated their state of anxiety more effectively than those in the negative future only and the reverse contrasting conditions. It seems then, regardless of whether mental contrasting refers to a positive or negative future, the order in which people imagine the future and the reality is essential for the effects of mental contrasting to occur (Oettingen, 2012; Kappes and Oettingen, 2014). That is, people need to design and imagine the future first, and only then elaborate the reality; only then can the future serve as an anchor for reality and the reality can adopt the meaning of an obstacle (Kappes et al., 2013).

We found a similar effect for the positive reality condition as for the mental contrasting condition. Apparently, reading and thinking about a feared future evokes enough salience of the feared future that subsequent thinking about the positive reality produces mental contrasting effects. That is, when the negative future is salient, the elaborated positive reality is interpreted against the background of the negative future. Thus, in the framework of an acute fear that is considered unlikely to materialize, sheer thinking about the positive reality was enough to regulate state anxiety arising from the feared future. In the present study, we had participants first read a section about the negative consequences of *E. coli* and subsequently imagine factors standing in the way that this future will in fact occur. This procedure corresponds to what participants do when they mentally contrast: juxtaposing thoughts about a negative future (i.e., suffering from *E. coli*) with a positive present reality (i.e., what stands in the way of *E. coli* spreading).

To sum up, in Study 1 we found anxiety-regulatory effects of mental contrasting of a negative future in the context of a bacterial epidemic. In Study 2 we wanted to conceptually replicate the findings in a different context. We sought to investigate the effects of mental contrasting on anxiety arising from idiosyncratic fears.

## Study 2: Regulation of Idiosyncratic Fears

In Study 2, we wanted to extend the findings of Study 1: whereas in Study 1 we defined the context of the fear (i.e., a bacterial epidemic), in Study 2 we tested the anxiety-regulatory function of mental contrasting in the broader context of an idiosyncratic fear. The ethical review committee of the University of Hamburg and New York University approved the procedure and materials.

Like Study 1, Study 2 was designed as an online study. We used a one-factorial (self-regulatory strategy: mental contrasting vs. negative future vs. positive reality vs. reverse contrasting) design. Participants were induced to apply the respective strategy to an idiosyncratic fear regarding the next 3 weeks of their lives. Based on the findings of Study 1, we hypothesized that participants in the mental contrasting and the positive reality condition would show less state anxiety regarding their fear-evoking event compared to the negative future and reverse contrasting conditions.

### Method

#### Participants

Based on the medium effect size observed in Study 1, we conducted power analysis for a one-way ANOVA with four groups to determine a sufficient sample size using an alpha of 0.05, a power of 0.80, and a medium effect size ($\eta_{p}^{2}$ = 0.06; Faul et al., 2013). The power analysis indicated that approximately 180 participants would be needed. To account for potential study dropouts, we recruited 218 participants via Amazon MTurk. Twelve participants were excluded because they did not write down their free thoughts and images during the self-regulatory procedure. Drop-outs were independent of condition. The remaining participants (*N* = 206) were included in the data analyses. All participants were living in the United States, 59% (*n* = 122) were female. Their age ranged from 18 to 81 years (*M* = 36.96, *SD* = 12.99).

#### Procedure and Materials

Participants were informed about the procedure of the study and signed the consent form. The study took about 20 min, and in the end, all participants were fully debriefed and received credit for their participation.

#### Idiosyncratic Fear

We wanted participants to apply the self-regulatory strategy to an idiosyncratic fear. Therefore, we first asked participants to name a fear-evoking event in their lives that they were facing within the next 3 weeks. Specifically, participants read:

Sometimes we are afraid of a negative future although we know our fears are unreasonable or unfounded. For example, there may be a specific future event or scenario that provokes fearful thoughts in you although you know it is very unlikely that your fears will bear out. Now, thinking about the next 3 weeks, please name an event or scenario that you will face that evokes such unfounded or unreasonable fears in you. Name an event or scenario, where you have such fears although you feel that they are unfounded or unreasonable.

Then participants were instructed to briefly name and write down their fear they associated with that event. For example, one participant named the fear of riding an escalator at the mall, although knowing that the escalators are reasonably safe. Another participant wrote of having to attend a party and being afraid of interacting with other people. Other examples participants mentioned included the fear of giving a presentation, the fear of traveling across the country, or the fear of going to the dentist.

#### Manipulation of the Self-Regulatory Strategy

Participants were randomly assigned to one of four conditions. The manipulation was in line with Study 1: in (1) the mental contrasting condition participants were asked “What is the worst thing that you associate with your feared future coming true? Identify the worst thing and write it down.” Subsequently, they were instructed to mentally elaborate on this aspect of the negative future. Specifically, participants read:

Now really think about this worst thing. Imagine the relevant events and scenarios as vividly as possible. Let your mind go! Do not hesitate to give your thoughts and images free rein. Take as much time and space as you need to write down your thoughts and images.

Afterward we asked participants to name the most important aspect of the present reality that was standing against their fear coming true:

Sometimes things do not happen although we are afraid they could. What is the most important aspect of the present reality that is standing against your feared future coming true? What should prevent your feared future from coming true? Identify the most important thing that should prevent that your feared future will actually happen and write it down.

Subsequently, they elaborated this aspect of the present reality and wrote their thoughts and images down. In sum, participants in the mental contrasting condition contrasted the negative future of their fear coming true with the present reality that was standing against their fear coming true. In (2) the negative future condition participants were asked to name and elaborate on the most important and the second most important aspects of the negative future of their fear coming true. In (3) the positive reality condition participants were only instructed to name and elaborate on the two most important aspects of the present reality that were standing against their fear coming true. In (4) the reverse contrasting condition participants were instructed to think about both the negative future and the positive reality. However, unlike in the mental contrasting condition participants in the reverse contrasting condition started with the positive reality and only then elaborated on the negative future.

#### State Anxiety

Right after the manipulation of the self-regulatory strategy we assessed state anxiety vis-à-vis their negative future as our primary dependent variable. We used the state anxiety subtest of the STAI like in Study 1. Internal consistency was high, Cronbach’s α = 0.96.

#### Control Variables

Like in Study 1, we controlled for participants’ individual trait anxiety using the STAI-T (Cronbach’s α = 0.86). To control for a baseline measure of state anxiety we used the same three items of the STAI-S like in the previous study (Cronbach’s α = 0.94). Subsequently, we assessed participants’ expectations to overcome their fear by asking “How likely do you think it is that you will be able to overcome your fear?,” and their incentive value to overcome their fear by asking “How important is it to you to overcome your fear?,” both items using a 7-point scale from 1 (*not at all*) to 7 (*very much so*). Finally, we assessed participants’ demographic data like gender, age, and profession.

### Results

#### Descriptive Data

Like in Study 1, we calculated sum scores for baseline trait anxiety (with a maximum possible score of 80) and baseline state anxiety (with a maximum possible score of 12). Participants had a moderate extent of trait anxiety (*M* = 38.97, *SD* = 11.99) and a moderate extent of baseline state anxiety (*M* = 8.32, *SD* = 2.45). When taking the means instead of the sum scores, the score of trait anxiety (38.97/20 = 1.95) seemed lower than both the score of baseline state anxiety (8.32/3 = 2.78) and the score of state anxiety as the dependent measure (52.66/20 = 2.63). These relatively high scores of state anxiety in Study 2 may be due to the fact that we asked participants to generate an idiosyncratic fear of the negative future as compared to Study 1, where we used a standardized negative future event in terms of an *E. coli* epidemic. Expectations and incentive value to overcome the fear were above the midpoint of the 7-point scale (expectations: *M* = 4.59, *SD* = 1.78; incentive value: *M* = 5.83, *SD* = 1.37).

#### State Anxiety

We computed a GLM with state anxiety as the dependent variable and condition as a fixed between-subjects factor. We included the continuous measures of trait anxiety, baseline state anxiety, expectations, and incentive value as covariates. Trait anxiety, *F*(1,198) = 9.87, *p* = 0.002, $\eta_{p}^{2}$ = 0.047, baseline state anxiety, *F*(1,198) = 48.26, *p* < 0.001, $\eta_{p}^{2}$ = 0.196, and expectations, *F*(1,198) = 5.13, *p* = 0.025, $\eta_{p}^{2}$ = 0.025, were all significant covariates. More importantly, we also found our hypothesized main effect of condition to be significant, *F*(3,198) = 10.90, *p* < 0.001, $\eta_{p}^{2}$ = 0.142.

**FIGURE 2:**
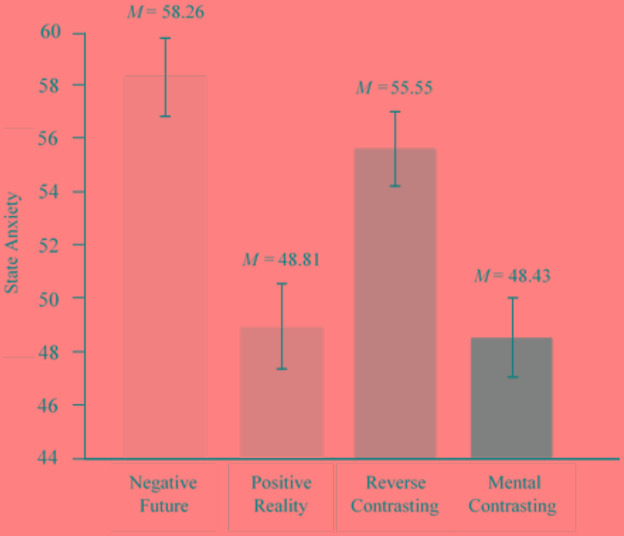
Effects of self-regulatory strategies on state anxiety vis-à-vis an idiosyncratic feared future. Error bars represent standard errors. Covariates appearing in the model are evaluated at the following values: Trait anxiety, *M* = 38.98; baseline state anxiety, *M* = 8.32; expectations, *M* = 4.59; and incentive value, *M* = 5.83.

Thus, depending on which self-regulatory strategy participants applied, they experienced a different extent of state anxiety. Participants who mentally contrasted the negative future with the present reality had the lowest state anxiety scores (*M* = 48.43, *SE* = 1.47, 95% CI [45.53, 51.34]), whereas participants who only reflected on the negative future had the highest state anxiety scores (*M* = 58.26, *SE* = 1.45, 95% CI [55.40, 61.12]), see **Figure 2**.

Pairwise comparisons revealed significant differences between the mental contrasting condition and the negative future condition, *t*(198) = 4.75, *p* < 0.001, $\eta_{p}^{2}$ = 0.102, 95% CI [-13.91, -5.74], and between the mental contrasting condition and the reverse contrasting condition, *t*(198) = 3.50, *p* = 0.001, $\eta_{p}^{2}$ = 0.058, 95% CI [-11.14, -3.10]. The pattern of results in the positive reality condition was similar to the mental contrasting condition. We found no differences between the mental contrasting condition and the positive reality condition, *t*(198) = 0.17, *p* > 0.05, $\eta_{p}^{2}$ = 0.00, 95% CI [-4.69, 3.94]. In fact, the positive reality condition showed significantly lower anxiety scores than the negative future condition, *t*(198) = 4.36, *p* < 0.001, $\eta_{p}^{2}$ = 0.088, 95% CI [-13.73, -5.18], and the reverse contrasting condition, *t*(198) = 3.15, *p* = 0.002, $\eta_{p}^{2}$ = 0.048, 95% CI [-10.97, -2.52].

An additional GLM without including covariates revealed the same results, *F*(3,202) = 10.14, *p* < 0.001, $\eta_{p}^{2}$ = 0.131. Pairwise comparisons revealed (1) significantly less state anxiety in the mental contrasting condition, than in the negative future condition, *t*(202) = 4.33, *p* < 0.001, $\eta_{p}^{2}$ = 0.085, 95% CI [-16.44, -6.15], and (2) significantly less state anxiety in the mental contrasting condition than in the reverse contrasting condition, *t*(202) = 4.07, *p* < 0.001, $\eta_{p}^{2}$ = 0.076, 95% CI [-15.42, -5.35]. There were no differences between the mental contrasting condition and the positive reality condition, *t*(202) = 0.46, *p* = 0.645, $\eta_{p}^{2}$ = 0.001, 95% CI [-6.71, 4.16]. We found no interaction effects between condition and any of the covariates all *ps* > 0.080.^2^

### Discussion

In Study 2, participants who were induced to mentally contrast a negative future with the positive present reality had lower state anxiety vis-à-vis that negative future than participants who either reflected on the negative future or reverse contrasted. In this regard, we replicated the anxiety regulatory effect of mental contrasting that we found in Study 1. Beyond that, we varied the context in which people applied the self-regulatory strategy: whereas in Study 1 we had participants think about a specific topic which we presented to them (i.e., a potential epidemic of *E. coli*), in Study 2 participants were asked to choose an idiosyncratic fear in their lives by themselves. These findings extend the results of Study 1 and underline the range of application in which people may benefit from mentally contrasting a negative future. Remarkably, whereas in Study 1 we found a medium effect size of condition, in Study 2 the effect size was large. This finding suggests that in the context of idiosyncratic fears it is particularly helpful to use mental contrasting for regulating state anxiety when facing a feared future.

Interestingly, like in Study 1, we found a similar anxiety-regulatory effect for the positive reality condition as for the mental contrasting condition. Participants who reflected on the positive reality showed less state anxiety than participants in the negative future and the reverse contrasting condition. We assume that by being instructed to name the fear in the beginning of the study, participants’ fear became highly salient. From a functional perspective, fear and anxiety play a key role in the organism’s defense system, and an effective defense must be quickly activated (Öhman, 2008). Consequently, “anxiety and worry are associated with an automatic processing bias” (Mathews, 1990, p. 462), oriented to threat. Thus, when participants are instructed to name an idiosyncratic fear, images and thoughts of the feared future immediately pop out. By thinking about a positive reality afterward, participants may inevitably pass a mental contrasting procedure. Our findings suggest that it is enough to mentally elaborate on the positive reality when the feared future is already salient. However, more research is required to reveal what exactly happens in the positive reality condition.

## General Discussion

In the present research, we investigated the effects of mental contrasting of a negative future on the regulation of state anxiety vis-à-vis that negative future. In two studies we found a beneficial effect of mental contrasting of a feared future on anxiety for different fear-evoking situations. In Study 1 participants who mentally contrasted a negative future of a potential bacterial epidemic with the positive reality standing against that feared future coming true, had less state anxiety than participants who either reflected on the negative future only, or who reverse contrasted. In Study 2, we demonstrated that mental contrasting of a negative future also leads to less state anxiety regarding the idiosyncratic fear.

In summary, our data suggest that mental contrasting of a negative future is a strategy to effectively cope with a variety of fears. People may use this strategy in a given moment to regulate their state anxiety. Of note is that mental contrasting does not change, but that it activates people’s expectations (Oettingen, 2012). Thus, in the present research we did not seek to enhance participants’ expectations that their fear would not come true, but to activate their expectations so that they imminently realize that their fear is unlikely to materialize.

One may argue that by mentally contrasting a negative future with the positive reality people experience a kind of mental desensitization. Systematic desensitization is a therapeutic approach that has been proven to be an effective component of the treatment of specific anxiety disorders by gradually exposing patients to their feared stimulus (e.g., Triscari et al., 2015). Thereby, people experience that their anxiety is unreasonable or at least disproportional to the actual feared stimulus. As a consequence, anxiety decreases. In mental contrasting of a negative future with the positive reality people are first mentally exposed to the fear evoking stimulus; however, mentally elaborating on the positive reality standing in the way of their fear coming true, people should realize that their fear is not in proportion with the actual danger which in turn should lead to a decrease in anxiety.

One may further argue that mental contrasting of a negative future with a positive reality may reduce risk-taking behavior. Specifically, by mentally contrasting a disproportionally feared future with a positive reality, the low probability of the negative future coming true is highlighted. As a consequence, people would be able to actively deal with the feared future without engaging in risk-taking behavior. For example, people who reduce their fear of *E. coli* would be less prone to risky behavior that might result from wanting to ward off getting infected (e.g., eating no fresh vegetables).

In both of our studies, we found a similar effect for the positive reality condition as for the mental contrasting condition. In other words, participants who were instructed to reflect on the positive reality only also exhibited less state anxiety than in the negative future condition and the reverse contrasting condition. As outlined above, we assume that by thinking about their fear and subsequently elaborating on the positive reality, participants in the positive reality condition passed a comparable procedure as participants in the mental contrasting condition. Even more, it raises the question if in the context of fears one cannot induce a true positive reality condition as far as participants should name their fear in the beginning of the study, because participants may automatically start to vividly imagine their feared future.

In contrast to the present results, Oettingen et al. (2005) did not find that participants in the positive reality condition showed mental contrasting effects. However, there was a crucial difference between our research and past research in the experimental manipulations of the positive reality condition: whereas Oettingen et al. (2005) actively guided participants away from the negative future (by having them reinterpret the validity of the negative future, thus having them focus only on the positive reality), our manipulation was in line with the classic instructions in the research on mental contrasting (i.e., in the positive reality condition, we had participants name and mentally elaborate on the most important and the second most important aspect of the positive reality; for a detailed description, see Oettingen et al., 2001; Oettingen, 2012). That is, in the positive reality condition of the present research, we did not have participants actively diminish the meaning of the negative future, and thus we did not prevent mental contrasting effects to occur.

In addition, because in the present research participants in the positive reality condition were instructed to think about the positive reality twice (to ensure that conditions were experimentally equivalent), one might argue that this “double dose” of positive reality should have caused them to show even less state anxiety than participants in the mental contrasting condition. But, remarkably, there were no differences between the positive reality condition and the mental contrasting condition. However, an advantage of mental contrasting over a positive reality condition is that in mental contrasting people are guided systematically through each stage of the contrasting procedure. This includes an explicit instruction to elaborate on both the negative future and the positive reality. In other words, mental contrasting is well structured and thus leads to predictable outcomes. In the positive reality condition, by contrary, there is no instruction to elaborate on the negative future. Research should shed light on how a salient feared future may produce mental contrasting effects.

Together with the findings by Oettingen et al. (2005), which showed that mental contrasting of negative, xenophobic fantasies in high school students increased tolerance toward foreign youth, the present research stands alone in investigating the effects of mental contrasting of a feared future to regulate state anxiety. Clearly, more research on this topic is needed. We also must point out that in both of our studies we explicitly confronted participants with a specific fear. Additionally, especially in Study 1, we confronted participants with a fear that they might have been thinking of only in the context of our study. Future research should find out whether mental contrasting leads to similar effects when participants are not experimentally prompted to face a specific feared future or asked to name a particular idiosyncratic feared future. Also, more research is needed in the context of a wide array of everyday life situations.

Research should also address the question of how to measure the dependent variables. The present research focuses on self-report measures of anxiety. However, to strengthen our findings, we suggest adding objective measures like physiological correlates of anxiety (e.g., fixation duration and saccade rates in eye-tracking measures, or muscle activation in electromyography, EMG) that are not subject to response biases. Physiological measures might be particularly helpful as Tichon et al. (2014) showed that there is correspondence between self-reported anxiety and physiological measures. Adding behavioral measures that are associated with worry cognitions and a lack of concentration would be helpful as well. For example, measuring performance at tasks that are impaired by worry cognitions or a lack of concentration (e.g., d2) would contribute to a better understanding of the effects of mental contrasting of a negative future with a positive reality.

### Mediating Processes

Research is also needed with respect to investigating potential mediating processes. Research on mental contrasting of a desired future has already demonstrated the mediating role of different cognitive and motivational processes (Oettingen et al., 2009; Kappes et al., 2012, 2013; Kappes and Oettingen, 2014). One might assume that these underlying mechanisms may be present also in mental contrasting of a negative future.

### Long-Term Effects

An important question is whether mental contrasting of a negative future attenuates anxiety in the long term. In our research, we used Spielberger’s STAI to assess participants’ state anxiety as our primary dependent variable. Thus, we focused on emotion regulation in the short term. In other words, we demonstrated short-term effects. The findings imply that people may use mental contrasting to reduce anxiety in a given situation. We assume that people who regularly apply mental contrasting with regard to state anxiety will achieve high regulatory competence in situations associated with high anxiety, and will eventually experience anxiety less frequently. As a result, we would expect trait anxiety to decrease. Research has shown that in fact, it is possible to reduce trait anxiety (e.g., Zettle, 2003). However, longitudinal studies are needed to provide insight into the effects of mental contrasting on trait anxiety.

### Dealing with Fears of Negative Futures That Are Likely to Occur

In our research we wanted participants to use mental contrasting of an unlikely negative future to down-regulate anxiety and thus to actively deal with it. However, there are situations in which a negative future is more likely to occur and in fact means a risk for people’s well-being. Similar to positive emotions that are shown not to be helpful for performance and well-being under all circumstances (Gruber et al., 2011), the reduction of anxiety seems not always to be helpful either. For example, it would obviously not be adequate to reduce a child’s fear of a busy road. As mentioned above, fear and anxiety have an important function in the organism’s defense system (Öhman, 2008). In other words, when a fear of a negative future is reasonable (because a negative future is likely to occur) and anxiety is proportional to the actual threat, it would be beneficial to avoid that negative future rather than approaching it. Thus, there are situations where a negative future needs to be avoided. However, even in these scenarios (i.e., if a negative future needs to be avoided) one may use the strategy of mental contrasting. In research by Oettingen et al. (2010), participants successfully set and pursued avoidance goals using mental contrasting of the negative future pertaining to continued cigarette consumption. Importantly, the experimental manipulation of mental contrasting regarding avoidance goals differs from the manipulation of mental contrasting regarding approach goals (i.e., the positive reality is framed as a potential loss rather than an obstacle; e.g., “when I continue to smoke, then I will lose a lot of money and cannot afford an extra holiday”; Oettingen et al., 2010). To sum up, mental contrasting can be used to build goals to approach a disproportionally feared future as well as to build goals to avoid a justifiably feared future.

## Conclusion

The presented research investigated the effects of mental contrasting of a negative future on state anxiety regarding that negative future. In two studies, we found that mental contrasting leads to less self-reported state anxiety regarding the feared future event. Participants who mentally elaborated on the positive reality also showed less state anxiety, probably due to a highly salient negative future. This effect and its mediating processes should be explored in future research. Whenever people find themselves in situations in which a fear prevents them from actively dealing with the future event (e.g., when facing a flight over the Atlantic, an exam, or a visit at the dentist), people may benefit from mental contrasting the negative future as it is a content-independent, time- and cost-effective self-regulation strategy that people can learn and apply by themselves to attenuate their anxiety.

## Ethics Statement

These studies were carried out in accordance with the recommendations of the “University Committee on Activities Involving Human Subjects” at New York University with informed consent from all subjects. All subjects gave informed consent in accordance with the Declaration of Helsinki. The Institutional Review Boards of New York University and the University of Hamburg approved the protocol and materials.

## Author Contributions

GO developed the study concept and both authors contributed to the study design and testing. Data collection, data analyses, and interpretation were performed by GB under the mentorship of GO. GB and GO drafted the manuscript and both authors approved the final version of the manuscript for submission.

## Conflict of Interest Statement

The authors declare that the research was conducted in the absence of any commercial or financial relationships that could be construed as a potential conflict of interest.

## Footnotes

1 There was a trend of an interaction effect between baseline state anxiety and self-regulatory strategy condition, *p* = 0.099. In the reverse contrasting condition, participants with a high baseline state anxiety showed a trend toward more state anxiety regarding the feared future.

2 There was a trend of an interaction effect between expectations and self-regulatory strategy condition, *p* = 0.081. In accord with past literature (Oettingen et al., 2005; see Oettingen, 2012), in the mental contrasting condition participants with high expectations to regulate their anxiety vis-à-vis an idiosyncratic feared future were somewhat more successful in reducing their state anxiety.
